# The Quorum-Sensing Regulator SdiA Activates *npsA* Expression and Modulates Cytotoxicity in *Klebsiella oxytoca*

**DOI:** 10.3390/microorganisms14051144

**Published:** 2026-05-19

**Authors:** Carlos J. Jiménez-Sánchez, Cristopher Perez, Sandra Rivera-Gutiérrez, Jorge Soria-Bustos, Fernando Chimal-Cázares, Roberto Rosales-Reyes, Santa Mejía-Ventura, Gabriela Hernández-Martínez, Miguel A. De la Cruz, Jorge A. Yañez-Santos, Maria L. Cedillo, James G. Fox, Miguel A. Ares

**Affiliations:** 1 Posgrado en Ciencias en Biomedicina y Biotecnología Molecular, Escuela Nacional de Ciencias Biológicas, Instituto Politécnico Nacional, Mexico City 11340, Mexico; cjimenezs1902@alumno.ipn.mx (C.J.J.-S.); cperezp1602@alumno.ipn.mx (C.P.); 2 Unidad de Investigación Médica en Enfermedades infecciosas y Parasitarias, Hospital de Pediatría, Centro Médico Nacional Siglo XXI, Instituto Mexicano del Seguro Social, Mexico City 06720, Mexico; chimal.fc@gmail.com (F.C.-C.); gabrielahmtz21@gmail.com (G.H.-M.); 3 Departamento de Microbiología, Escuela Nacional de Ciencias Biológicas, Instituto Politécnico Nacional, Mexico City 11340, Mexico; srivera@ipn.mx; 4 Centro de Detección Biomolecular, Benemérita Universidad Autónoma de Puebla, Puebla 72592, Mexicomiguel.delacruzv@correo.buap.mx (M.A.D.l.C.); jorge.yanez@correo.buap.mx (J.A.Y.-S.); lilia.cedillo@correo.buap.mx (M.L.C.); 5 Unidad de Medicina Experimental, Facultad de Medicina, Universidad Nacional Autónoma de México, Mexico City 04510, Mexico; rrosalesr@ciencias.unam.mx; 6 Facultad de Medicina, Benemérita Universidad Autónoma de Puebla, Puebla 72410, Mexico; 7 Division of Comparative Medicine, Massachusetts Institute of Technology, Cambridge, MA 02139, USA; jgfox@mit.edu

**Keywords:** *Klebsiella oxytoca*, *npsA*, gene expression, SdiA, quorum-sensing, cytotoxicity

## Abstract

Toxigenic *Klebsiella oxytoca* strains linked to antibiotic-associated hemorrhagic colitis produce the cytotoxins tilimycin and tilivalline, which contribute to intestinal epithelial damage during infection. Tilimycin and tilivalline are synthesized by enzymes encoded within the nonribosomal peptide synthetase (NRPS) operon, yet the regulatory mechanisms controlling operon expression remain poorly understood. SdiA, an orphan LuxR-type quorum-sensing regulator, detects exogenous *N*-acyl homoserine lactones (AHLs) produced by neighboring bacterial species and modulates gene expression in response to interspecies communication. Although SdiA has been implicated in virulence regulation in several enteric pathogens, its role in *K. oxytoca* remains unclear. This study demonstrates that SdiA positively regulates *npsA*, the first gene in the NRPS operon, and that this regulatory effect is enhanced in the presence of exogenous AHL. Electrophoretic mobility shift assays indicate that SdiA directly binds to the upstream regulatory region of *npsA*, supporting a direct interaction consistent with positive transcriptional regulation. Furthermore, deletion of *sdiA* significantly reduces cytotoxicity toward HeLa cells under the conditions tested. Collectively, these findings identify SdiA as a quorum-sensing-responsive activator of *npsA* expression and support its role in modulating cytotoxicity in toxigenic *K. oxytoca* strains. These results provide new insight into the influence of interspecies quorum-sensing signals on virulence-associated regulatory pathways in *K. oxytoca*.

## 1. Introduction

*Klebsiella oxytoca* is a Gram-negative bacterium that forms part of the human gut microbiota. It colonizes approximately 2–10% of healthy individuals [1,2]. Although typically regarded as a commensal organism, *K. oxytoca* can behave as an opportunistic pathogen and is associated with a range of healthcare-related infections. Notably, it exhibits intrinsic resistance to β-lactam antibiotics due to chromosomally encoded β-lactamases [3]. Perturbation of the intestinal microbiota during antibiotic treatment can lead to dysbiosis. This reduces colonization resistance and facilitates the expansion of pathobionts such as *K. oxytoca* [4].

A subset of *K. oxytoca* strains produces enterotoxins responsible for antibiotic-associated hemorrhagic colitis (AAHC). This condition is identified by abdominal pain and bloody diarrhea [5]. The enterotoxins tilimycin (TM) and tilivalline (TV) are synthesized by a nonribosomal peptide synthetase (NRPS) system encoded by the NRPS operon within the *til* pathogenicity island and specifically constituted by the *npsA*, *thdA*, and *npsB* genes [6]. TM induces DNA damage and arrests the cell cycle at the G1/S transition, while TV stabilizes microtubules and blocks progression at the G2/M phase. Both toxins compromise epithelial barrier integrity by altering tight junction proteins, such as claudin-1, leading to apoptosis and intestinal damage [4,7]. Although the effects of TM and TV are well characterized, the regulatory mechanisms controlling NRPS operon expression remain poorly understood.

Quorum-sensing (QS) is a bacterial communication system that enables coordinated regulation of gene expression in response to population density. QS depends on the production, release, accumulation, and detection of diffusible signaling molecules called autoinducers. As bacterial cell density increases, the concentration of autoinducers in the environment also rises, thereby modulating gene expression at the population level. In Gram-negative bacteria, QS is mostly mediated by *N*-acyl homoserine lactones (AHLs), which are small, diffusible molecules synthesized by LuxI-type enzymes. These signals freely diffuse across the cell membrane and, at sufficiently high concentrations, are recognized by cognate LuxR-type transcriptional regulators. In many LuxR-type systems, AHL binding induces conformational changes that can modulate DNA binding and transcriptional regulation of target genes [8,9,10].

The detection of AHLs in the gut has been debated due to the methods and the chemical instability of these molecules [11]. Initial studies with LuxR biosensors reported low or undetectable AHL levels. Later, mass spectrometry confirmed the presence of a range of AHLs in intestinal samples from both healthy and dysbiotic individuals [12]. Also, the detection of LuxI/LuxR-like genes in gut bacteria suggests endogenous AHL production [11].

AHL availability within the gut is likely heterogeneous. It is influenced by factors such as pH, enzymatic degradation, and the spatial distribution of microbial populations [11]. In this complex environment, some bacteria lack AHL synthases but possess LuxR-type receptors, such as SdiA. These are found in *Escherichia*, *Enterobacter*, and *Klebsiella.* They detect external AHL signals and modulate gene expression [13].

Detection of signals from nearby bacteria is significant in the gut microbiota, where many species coexist. Acyl-homoserine lactones (AHLs) act as signals between different kingdoms. They affect the host by maintaining intestinal balance and controlling inflammation [13,14]. Here, SdiA may act as a central sensor. It detects AHLs and regulates genes, including those in virulence pathways such as *npsA*, the first gene of the NRPS operon in *K. oxytoca*.

Although interspecies signaling is increasingly recognized in bacterial pathogenesis, the regulatory connection between quorum sensing and toxin biosynthesis in *K. oxytoca* remains unresolved. To address this gap, this study demonstrates that the orphan LuxR-type regulator SdiA positively regulates *npsA* expression and directly binds its upstream regulatory region. Genetic, transcriptional, and biochemical analyses reveal that SdiA positively regulates *npsA* by binding directly to its upstream regulatory region, a process significantly potentiated by the presence of AHL.

These findings suggest that *K. oxytoca* integrates quorum-sensing signals from neighboring microbes to regulate *npsA* expression and associated cytotoxic phenotypes, with SdiA acting as the interface that links these microbial signals to harmful effects in hosts. This provides insight into regulatory mechanisms potentially associated with AAHC and highlights interspecies quorum-sensing as a potential target for managing toxin-mediated disease.

## 2. Materials and Methods

### 2.1. Bacterial Strains

Experimental procedures were conducted using the toxigenic wild-type (WT) strain *K. oxytoca* MIT 09-7231, a clinical isolate from mouse tumor abscess. This WT strain served as the parental strain for the isogenic ∆*sdiA* mutant and its trans-complemented counterpart (∆*sdiA* pT3-SdiA), both of which were included in the study. The WT strain was previously characterized by API 20E profiling (bioMérieux, Marcy l’Etoile, France), amplification of the polygalacturonase-encoding *pehX* gene, and 16S rRNA gene sequencing [15]. Table 1 summarizes the characteristics of all strains and plasmids.

### 2.2. Growth Condition

Cells were routinely grown in tryptic soy broth (TSB; Difco, Beirut, Lebanon) at 37 °C under aerobic conditions. Growth media contained ampicillin (100 µg/mL), kanamycin (50 µg/mL), or tetracycline (10 µg/mL) (Sigma-Aldrich, St. Louis, MO, USA) as needed. For all assays, *K. oxytoca* cells were harvested at late stationary phase with an OD_600_ of about 1.6.

In selected experiments, cultures were supplemented with *N*-(3-oxo-octanoyl)-L-homoserine lactone (AHL) to evaluate its effects. AHL was dissolved in chloroform and added to the culture medium before bacterial inoculation, at the start of subculture from the overnight culture, to achieve a final concentration of 10 µM. This specific AHL and concentration were selected based on previous research indicates that SdiA homologs preferentially recognize medium-chain oxo-substituted AHLs, especially *N*-(3-oxo-octanoyl)-L-homoserine lactone, which serves as one of the most effective ligands for SdiA-mediated activation in *Salmonella enterica* [20]. Thus, these selections ensure the experimental conditions effectively probe the relevant signaling pathway.

In the same study, dose–response analyses demonstrated that SdiA-dependent activation is concentration-dependent and approaches saturation near 10 µM for several AHL molecules [20]. Consistent with these findings, micromolar concentrations of AHLs are routinely used in in vitro quorum-sensing assays, ensuring sufficient receptor occupancy under controlled conditions [21,22,23,24,25]. To control vehicle effects, samples received an equivalent volume of chloroform, resulting in a final concentration of 0.1% (*v*/*v*). This concentration was chosen based on prior research indicating that 0.1% chloroform does not induce cytotoxicity in epithelial cells [26]. Inclusion of this solvent control revealed no significant effect of chloroform on bacterial growth, HeLa cell viability, or baseline LDH release.

### 2.3. Generation of the ΔsdiA Mutant Strain

The *sdiA* gene in *K. oxytoca* was deleted using the λ-Red recombinase system, following a previously described one-step mutagenesis protocol [18]. A PCR fragment was generated using gene-specific primers (Table S1). This fragment contained a kanamycin resistance cassette flanked by sequences homologous to the upstream and downstream regions of the *sdiA* locus. The purified DNA products were introduced by electroporation into competent *K. oxytoca* cells carrying the λ-Red recombinase helper plasmid pKD119. Recombinase expression was induced by adding 1% (*w*/*v*) L-(+)-arabinose (Sigma-Aldrich, St. Louis, MO, USA). Successful replacement of the target gene and chromosomal integration of the resistance marker were confirmed by PCR analysis and DNA sequencing. Once mutant strains were established, plasmids were constructed for complementation and protein expression studies.

### 2.4. Plasmid Construction

Complementation of the Δ*sdiA* mutant strain used the pT3-SdiA expression plasmid. The *sdiA* coding sequence from *K. oxytoca* was amplified by PCR using the primers listed in Supplementary Table S1. After purification, the amplicon was digested with KpnI and BamHI and ligated into the pMPM-T3 vector. For recombinant SdiA protein production and purification, a modified *sdiA* variant with an N-terminal hexahistidine tag (His_6_-tag) was engineered. This His_6_-*sdiA* fragment was cloned into the pMPM-T6 vector using NcoI and HindIII restriction sites. The resulting construct was pT6-SdiA. Assembly and sequence fidelity of all plasmids were verified by DNA sequencing. With these plasmids generated, molecular and protein assays were pursued.

### 2.5. RNA Extraction and Reverse Transcription–Quantitative PCR Analysis

Total RNA was isolated from *K. oxytoca* cells harvested at the stationary growth phase (OD_600_ = 1.6) using the hot phenol method [27]. Contaminating genomic DNA was removed by digestion with the TURBO DNA-free Kit (Invitrogen, Waltham, MA, USA). RNA yield and purity were quantified with a NanoDrop ONE spectrophotometer (Thermo Fisher Scientific, Waltham, MA, USA). Sample integrity was checked by visualization on 1.5% bleach-denaturing agarose gels [28]. This RNA served as the template for downstream Reverse Transcription-quantitative PCR (RT-qPCR).

First-strand complementary DNA (cDNA) was synthesized from 1 µg of total RNA using the RevertAid First Strand cDNA Synthesis Kit (Thermo Fisher Scientific, Waltham, MA, USA) according to the manufacturer’s protocol. Negative controls lacking reverse transcriptase were included in each assay. These cDNA preparations were used for Quantitative PCR (qPCR).

The qPCR was performed on a LightCycler 480 instrument (Roche Diagnostics, Basel, Switzerland) using SYBR Green I Master Mix (Roche Diagnostics, Basel, Switzerland). Amplification mixtures (10 µL total volume) contained 5 µL of 2X SYBR Green I Master Mix, 2.5 µL of synthesized cDNA (approximately 25 ng), 1.5 µL of nuclease-free water, and 0.5 µL each of forward and reverse primers (20 µM; see Supplementary Table S1). The results from these reactions were analyzed for methodological rigor and reliability.

qPCR assays were performed in technical triplicate. Samples from three independent biological replicates were used. The *rrsH* transcript served as the endogenous reference for normalization of gene expression. Cycling conditions were an initial denaturation at 95 °C for 10 min, followed by 45 cycles of 95 °C for 10 s, 59 °C for 10 s, and 72 °C for 10 s. Fluorescence was measured at the end of each cycle. Melt-curve analysis confirmed specificity, using 10 s at 95 °C, a ramp from 65 °C to 97 °C, and a final 10 s at 40 °C.

Methodological rigor was ensured by including no-template and no-reverse transcriptase controls. Relative fold changes in gene expression were calculated using the 2^−∆∆Ct^ method [29,30]. Data represent the average values from three independent biological assays.

### 2.6. Purification of Recombinant His_6_-SdiA Protein

For recombinant protein production used in DNA-protein interaction assays with the *npsA* gene promoter, the pT6-SdiA expression plasmid was introduced into chemically competent *E. coli* BL21 (DE3) cells. Transformants were selected on Lysogeny Broth (LB) agar containing the appropriate antibiotic. Individual colonies were inoculated into LB with tetracycline (10 µg/mL) and incubated overnight at 37 °C. Subsequently, 2 mL of this culture was transferred into 200 mL of Terrific Broth with the same antibiotic and incubated at 37 °C with shaking until OD_600_ reached 0.4. Expression of His_6_-SdiA was induced by adding L(+)-arabinose (Sigma-Aldrich, St. Louis, MO, USA) to 0.1% (*w*/*v*) at 37 °C for 5 h. Induced cells were then harvested for protein extraction and purification.

Biomass was harvested by centrifugation at 12,000× *g* for 10 min. For extraction under denaturing conditions, the cell pellet was resuspended in 1X Phosphate-Buffered Saline (PBS) (pH 8.0) containing 8 M urea and 0.3 M NaCl. Cells were disrupted by sonication on ice for 20 min, with 1 min pulses separated by 1 min of cooling. The crude lysate was clarified by centrifugation at 16,000× *g* for 20 min at 4 °C. The resulting supernatant was filtered through a 0.2 µm filter. This extract was then subjected to affinity chromatography to isolate the His_6_-SdiA protein.

For affinity chromatography, the clarified extract was loaded onto a pre-equilibrated nickel-nitrilotriacetic acid (Ni-NTA) agarose column (Qiagen, Hilden, Germany). Contaminants were removed by sequential washing steps (10 mL each) with the base buffer (1X PBS, 8 M urea, 0.3 M NaCl; Sigma-Aldrich, St. Louis, MO, USA) containing increasing imidazole concentrations: 5, 10, 15, and 25 mM. The recombinant protein was then eluted using 6 mL of the same buffer with 250 mM imidazole. Eluted fractions were analyzed and prepared for further use.

Collected fractions were analyzed using 12% sodium dodecyl sulfate-polyacrylamide gel electrophoresis (SDS-PAGE) and stained with Coomassie Brilliant Blue R-250 (Bio-Rad Protein Assay, Hercules, CA, USA). Pooled eluates were dialyzed at 4 °C with gentle agitation using a MWCO 12 kDa cellulose membrane (Sigma-Aldrich, St. Louis, MO, USA) to remove urea and imidazole, thereby facilitating protein refolding. Renaturation was achieved by dialyzing for 12 h against 1 L of buffer containing 20 mM Tris-HCl at pH 8.0, 50 mM KCl, 1 mM DTT, and 10% *v*/*v* glycerol (Sigma-Aldrich, St. Louis, MO, USA). Urea concentrations were sequentially reduced from 6 M to 0 M (6 M, 4 M, 2 M, 1 M, 0.5 M, 0 M), with buffer exchanged every 2 h to match the decreasing urea concentration [31,32]. This buffer composition closely approximates physiological ionic conditions, which are critical for SdiA folding. The addition of glycerol further improved protein stability and minimized aggregation during renaturation. The dialyzed and refolded protein was subsequently prepared for quantification and storage.

After dialysis, samples were centrifuged at 12,000× *g* for 10 min at 4 °C to remove insoluble aggregates. The supernatant containing soluble, renatured His_6_-SdiA was collected. Protein concentration was determined using the Bradford colorimetric method (Bio-Rad Protein Assay, Hercules, CA, USA) with bovine serum albumin (BSA; Sigma-Aldrich, St. Louis, MO, USA) as the standard. For long-term storage, purified protein was aliquoted and stored at −70 °C in stabilization buffer (50% *v*/*v* glycerol, 10 mM Na_2_HPO_4_, 1.8 mM KH_2_PO_4_, 137 mM NaCl, 2.7 mM KCl, pH 7.4; Sigma-Aldrich, St. Louis, MO, USA). The purified protein was then used in subsequent functional interaction studies.

### 2.7. Electrophoretic Mobility Shift Assays

Electrophoretic mobility shift assays (EMSA) were performed to assess the binding of recombinant His_6_-SdiA protein to the regulatory region of the *npsA* gene. DNA probes covering the regulatory regions of *K. oxytoca npsA* and *E. coli ftsQ* (positive control) [33] and an internal fragment of *K. oxytoca pehX* (negative control) were amplified by PCR from genomic DNA. Purified DNA probes were standardized to 300 fmol per reaction. These preparations enabled a detailed investigation of SdiA-DNA binding parameters.

Binding reactions were performed in 20 µL volumes containing 1X binding buffer (20 mM Tris-HCl, pH 7.5; 50 mM KCl; 2.0 mM MgCl_2_; 0.1 mM EDTA; 1.0 mM DTT; 0.01% *v*/*v* Tween-20; 2% *v*/*v* glycerol; 0.1 mg/mL BSA; Sigma-Aldrich, St. Louis, MO, USA). *N*-(3-Oxo-octanoyl)-L-homoserine lactone (AHL; Sigma-Aldrich, St. Louis, MO, USA) was added at a final concentration of 10 µM. AHL was included based on previous reports indicating that LuxR-family regulators can respond to exogenous AHL signals during transcriptional regulation. Purified His_6_-SdiA was added at increasing concentrations (0.00, 0.25, 0.30, 0.35, and 0.40 µM). Each sample was then incubated with the DNA probe for 20 min at room temperature.

Samples were resolved by electrophoresis on 6% non-denaturing polyacrylamide gels in 0.5X Tris-borate-EDTA (TBE) buffer at 120 V. Temperature was controlled to maintain complex stability. Gels were stained with ethidium bromide (0.5 µg/mL), rinsed with distilled water, and visualized by UV transillumination. SdiA-DNA complexes appeared as retarded bands, while free DNA probe bands migrated more rapidly. These results supported a direct regulatory role for SdiA on the *npsA* gene. Further validation of binding sites was conducted using an in silico analysis as described below.

### 2.8. Identification of a Promoter and SdiA-Binding Motif in the npsA Regulatory Region

Promoter prediction within the *npsA* regulatory region was performed by analyzing 500 nucleotides upstream of the start codon. The *K. oxytoca* genomic sequence (GenBank accession no. GCF_001078175.1) was used for this analysis. The Neural Network Promoter Prediction web tool (https://fruitfly.org/seq_tools/promoter.html (accessed on 31 March 2026)) was also employed.

Potential SdiA-binding sites in the *npsA* gene regulatory region were identified through in silico sequence analysis using the PRODORIC v2.0 web database (https://www.prodoric.de/ (accessed on 31 March 2026)). A 500-nucleotide region upstream of the *npsA* translational start codon (ATG) was extracted for further analysis. Motif searches and comparisons were performed using the Virtual Footprint tool in PRODORIC. Predictive alignments were based on the established SdiA consensus recognition sequence (5′-AAAAG(N8)GAAAA-3′), previously identified in related *Enterobacteriaceae* [33,34,35,36]. These analyses supported the experimental EMSA results. They also increased confidence in the identification of the SdiA binding site.

### 2.9. LDH Cytotoxicity Assays

Cytotoxicity was evaluated by measuring lactate dehydrogenase (LDH) release using the CyQUANT LDH Cytotoxicity Assay Kit (Invitrogen, Waltham, MA, USA), following the manufacturer’s instructions (https://documents.thermofisher.com/TFS-Assets%2FLSG%2Fmanuals%2FMAN0018500_CyQUANT-LDH-Cytotoxicity-Assay-Kit_PI.pdf (accessed on 31 March 2026)). HeLa cells were maintained in Dulbecco’s Modified Eagle Medium (DMEM) supplemented with 4.5 g/L glucose and 10% fetal bovine serum (FBS) (Gibco, Waltham, MA, USA). Cells were seeded in 96-well flat-bottom plates at a density of 1 × 10^4^ cells. Cell viability and counts were previously determined via trypan blue exclusion using a Neubauer chamber [37].

For the cytotoxicity assay, 90 µL of HeLa cell suspension was treated with 10 µL of cell-free filtered supernatants obtained from the WT, Δ*sdiA*, and Δ*sdiA* pT3-SdiA strains. As an additional control, the previously generated Δ*npsA* mutant strain from this research group [16], which lacks the ability to produce the TM and TV cytotoxins, as previously demonstrated [6,38], was included. The use of this mutant strain confirmed that the cytotoxic effects observed in the assay were specifically associated with TM and TV production by *K. oxytoca*. All bacterial strains were pre-cultured in the absence or presence of 10 µM AHL prior to supernatant collection. Plates were subsequently incubated for 48 h at 37 °C in a humidified atmosphere containing 5% CO_2_, conditions previously established as optimal for detecting measurable cytotoxic effects [15,16,39,40,41].

Following incubation, 50 µL of the supernatant from each well was transferred to a fresh 96-well plate, and LDH activity was quantified by adding the respective kit reagents. Absorbance was recorded at 490 nm and 680 nm using a Multiskan Ascent microplate reader (Thermo Fisher Scientific, Waltham, MA, USA). The final LDH release was calculated as the difference between these two optical density (OD) values. Phosphate-buffered saline PBS and tryptic soy broth TSB were used as negative controls, whereas the provided lysis buffer served as the positive control. All assays were performed in triplicate across three independent biological replicates.

### 2.10. Statistical Analyses

All statistical analyses were conducted using GraphPad Prism software, version 10.5.0 (GraphPad Software, San Diego, CA, USA). Differences between experimental groups were assessed using one-way analysis of variance (ANOVA) followed by Tukey’s multiple-comparison post hoc test. Statistical significance was set at *p* < 0.05.

## 3. Results

### 3.1. SdiA Activates the Expression of the npsA Gene

**Figure 1:**
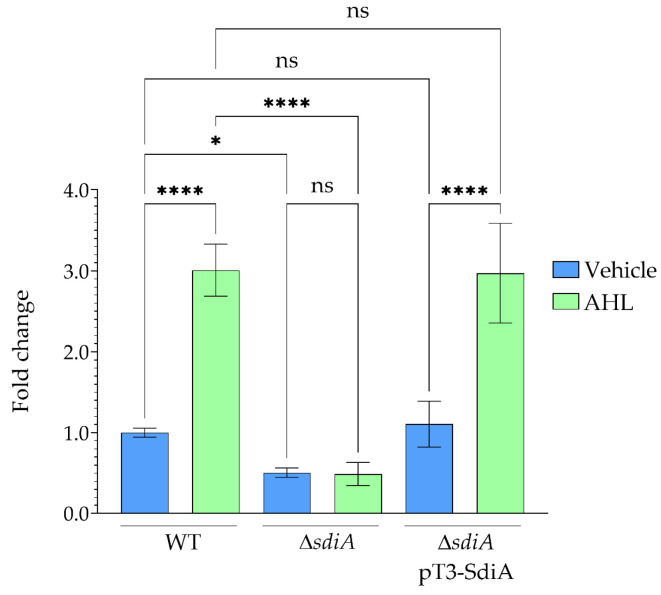
Regulation of *npsA* expression by SdiA in *K. oxytoca.* Relative *npsA* transcript levels were quantified in wild-type (WT), mutant (∆*sdiA*), and complemented (∆*sdiA* pT3-SdiA) strains cultured in tryptic soy broth (TSB) with chloroform as vehicle and with *N*-(3-Oxo-octanoyl)-L-homoserine lactone (AHL) at a final concentration of 10 µM. Results are presented as mean ± standard deviation from three independent biological replicates. Statistical significance was determined relative to WT using one-way ANOVA with Tukey’s multiple comparison test (* *p* < 0.05; **** *p* < 0.0001; ns, not significant).

The effect of SdiA on *npsA* gene expression was analyzed via RT-qPCR in WT, Δ*sdiA* mutant, and Δ*sdiA*pT3-SdiA complemented *K. oxytoca* strains. In the WT strain, addition of AHL led to a distinct increase in *npsA* expression, resulting in a 3-fold induction compared to the vehicle control (Figure 1). This identifies AHL-dependent activation of *npsA* as a key result in the WT background.

Deletion of *sdiA* resulted in a substantial reduction in *npsA* expression, as the Δ*sdiA* mutant exhibited approximately a 2-fold decrease compared to the WT strain under basal conditions. Notably, *npsA* expression in the Δ*sdiA* mutant did not change following AHL supplementation, indicating that SdiA is required for AHL responsiveness.

Thus, the disparity in *npsA* expression between WT and Δ*sdiA* mutant is more pronounced under AHL-treated conditions, reflecting induction exclusively in the WT strain (Figure 1). Together, these results indicate that SdiA mediates AHL-responsive activation of *npsA* transcription.

Moreover, complementation of the Δ*sdiA* mutant with pT3-SdiA restored *npsA* expression to WT levels under both AHL-supplemented and vehicle control conditions (Figure 1), confirming that the observed changes in gene expression are specifically due to the loss of *sdiA*. These findings indicate that SdiA positively contributes to both basal and AHL-induced *npsA* expression, establishing its central role in signal-dependent regulation.

No significant differences in bacterial growth were detected among the strains or experimental conditions. The reference gene *rrsH* exhibited stable expression across all conditions (data not shown). These findings support the reliability of RT-qPCR normalization and validate the observed differences in *npsA* expression.

### 3.2. Identification of Putative Promoter and SdiA-Binding Site in the npsA Regulatory Region

The cytotoxin biosynthetic genes are organized within the NRPS (nonribosomal peptide synthetase) operon, which comprises *npsA*, *thdA*, and *npsB* [6,38]. In silico analyses examined the regulatory architecture of this operon, with particular attention to potential promoter elements and SdiA-binding sites located upstream of *npsA*.

**Figure 2:**
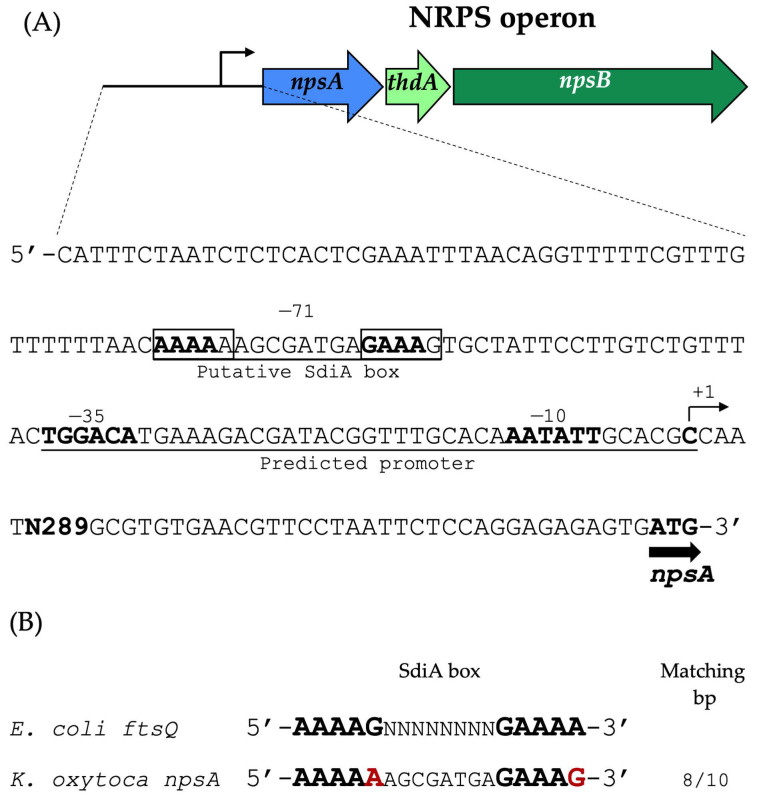
In silico characterization of the NRPS operon regulatory region. (**A**) The schematic shows the genetic organization of the NRPS operon and the upstream regulatory region of *npsA*. The start codon (ATG) appears in bold. The predicted promoter region is indicated. The −35 and −10 elements are both bolded and underlined. The transcription start site (+1) is displayed in bold. A putative SdiA-binding box at the −71 position is presented within the regulatory region and is underlined. Nucleotides predicted to be important for SdiA recognition are boxed, with those matching the consensus sequence shown in bold. (**B**) Sequence alignment of the predicted SdiA box identified upstream of *npsA* in *K. oxytoca* with the established SdiA box sequence from *ftsQ* in *E. coli*. Variations relative to the consensus are highlighted in bold red.

A putative promoter was predicted 329 bp upstream of the *npsA* coding sequence. Additionally, a candidate SdiA-binding box was identified within the same regulatory region, located 71 bp upstream of the predicted transcription start site (+1) (Figure 2).

The spatial arrangement of these elements suggests a regulatory mechanism in which SdiA binds to the DNA sequence immediately upstream of the promoter region. The predicted regulatory arrangement supports a potential role for SdiA in the positive regulation of *npsA* expression. Further experimental studies are required to clarify the precise molecular mechanism by which SdiA modulates promoter activity, such as through site-directed mutagenesis of the putative SdiA-binding site within the *npsA* regulatory region.

### 3.3. SdiA Directly Interacts with npsA Regulatory Region

**Figure 3:**
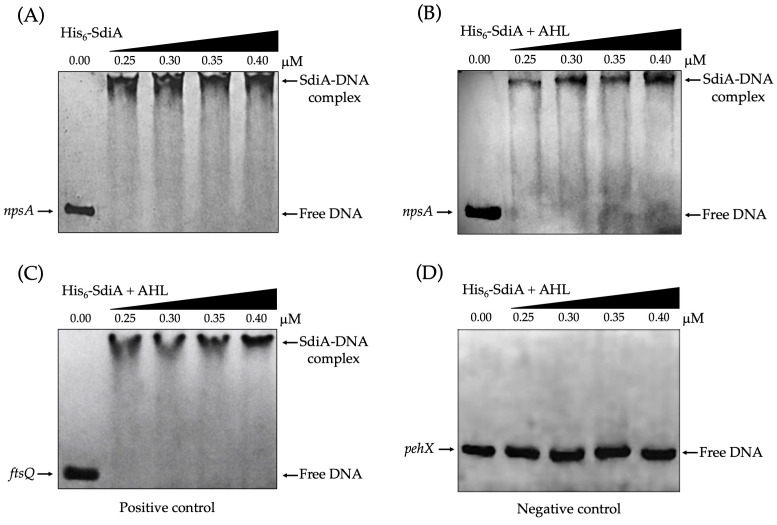
SdiA specifically binds to the *npsA* regulatory region. Electrophoretic mobility shift assays (EMSA) were performed using increasing concentrations of purified His_6_-SdiA to assess DNA binding. (**A**) Binding of His_6_-SdiA to the upstream regulatory region of *npsA* in the absence of *N*-(3-Oxo-octanoyl)-L-homoserine lactone (AHL) at a final concentration of 10 µM. (**B**) Binding of His_6_-SdiA to the same region in the presence of AHL. In both conditions, a concentration-dependent shift in DNA mobility is observed, indicating formation of SdiA–DNA complexes. (**C**) Binding of His_6_-SdiA to the promoter region of *ftsQ* from *E. coli*, used as a positive control. (**D**) No shift is observed using a DNA fragment corresponding to the coding region of *pehX* from *K. oxytoca*, used as a negative control. Arrows indicate free DNA and SdiA–DNA complexes. DNA was visualized by ethidium bromide staining.

To assess direct interaction between SdiA and the *npsA* regulatory region, recombinant His_6_-tagged SdiA was purified and subjected to EMSA. The assays revealed a clear, reproducible, and concentration-dependent DNA mobility retardation, indicating stable SdiA–DNA complex formation. This mobility shift was observed in the absence (Figure 3A) and presence (Figure 3B) of AHL, demonstrating that SdiA binds the *npsA* upstream regulatory region in vitro under both conditions.

While SdiA-mediated activation of *npsA* expression increases in the presence of AHL during bacterial growth, EMSA experiments indicate that SdiA binds the *npsA* regulatory region in vitro regardless of exogenous AHL.

The specificity of the SdiA–DNA interaction was validated using both positive and negative controls. The regulatory region of *ftsQ* from *E. coli*, a well-characterized SdiA target, exhibited the expected mobility shift (Figure 3C). Conversely, a DNA fragment from the coding region of *pehX* in *K. oxytoca*, used as a negative control, did not display a shift under identical conditions (Figure 3D), confirming sequence-specific interaction. Collectively, these results demonstrate that SdiA directly and specifically binds the *npsA* promoter region.

### 3.4. SdiA Enhances the Cytotoxic Activity of K. oxytoca on Epithelial Cells

**Figure 4:**
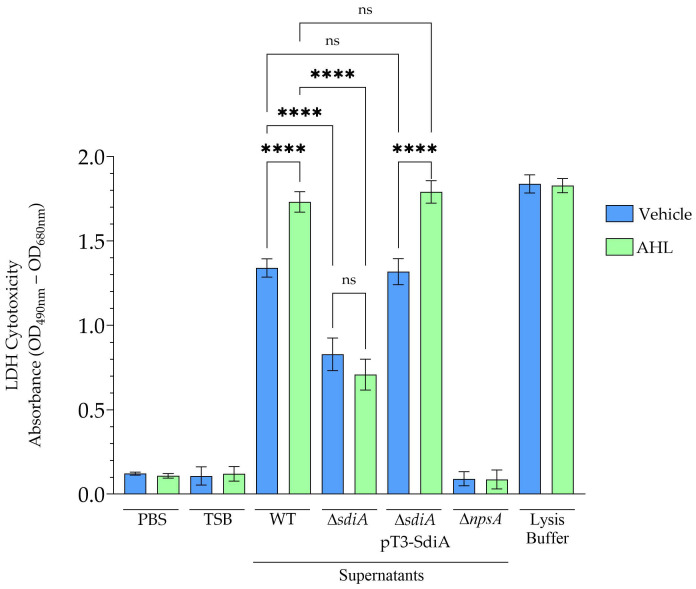
Assessment of cytotoxicity in *K. oxytoca* strains by lactate dehydrogenase (LDH) assay. HeLa cells were incubated for 48 h with either tryptic soy broth (TSB) alone or cell-free supernatants from different *K. oxytoca* strains: WT, mutant ∆*sdiA*, complemented ∆*sdiA* pT3-SdiA, and mutant ∆*npsA*. Each strain was grown to an OD_600_ of 1.6, with 0.1% chloroform as vehicle and with *N*-(3-Oxo-octanoyl)-L-homoserine lactone (AHL) at a final concentration of 10 µM. Cytotoxicity was measured by quantifying extracellular LDH release. Baseline and maximum LDH levels were set using phosphate-buffered saline PBS and lysis buffer, respectively. Statistical significance is shown as follows: **** *p* < 0.0001; ns, not significant.

The impact of SdiA on *K. oxytoca* cytotoxicity was assessed using cell-free supernatants from the toxigenic WT, the ∆*sdiA* mutant, and the complemented ∆*sdiA* pT3-SdiA strain. Cytotoxicity was measured by lactate dehydrogenase (LDH) release in HeLa cells. Supernatants from the WT strain induced substantial cytotoxicity, resulting in pronounced HeLa cell death. This effect was significantly enhanced when bacteria were cultured in the presence of AHL (Figure 4), indicating that quorum-sensing signals potentiate toxin-associated activity.

Supernatants from the ∆*sdiA* mutant exhibited a marked reduction in cytotoxic activity under both tested conditions, regardless of AHL presence. The absence of *sdiA* significantly reduced the cytotoxic phenotype observed under the tested conditions. In contrast, cytotoxicity in the complemented ∆*sdiA* pT3-SdiA strain was restored to WT levels, confirming that the phenotype is specifically associated with the loss of SdiA (Figure 4). The solvent control did not significantly affect bacterial growth, HeLa cell viability, or basal LDH release under the experimental conditions evaluated (data not shown).

To further confirm the association between *npsA* and the cytotoxic phenotype observed in this study, supernatants from the ∆*npsA* mutant strain were analyzed as a negative control. Under the experimental conditions tested, these supernatants did not induce detectable HeLa cell death, consistent with previous reports of reduced epithelial cytotoxicity in *npsA*-deficient strains [6,38]. Collectively, these findings indicate that SdiA modulates the cytotoxic phenotype in *K. oxytoca*.

## 4. Discussion

The quorum-sensing regulator SdiA acts as a positive regulator of *npsA*, the initial gene in the NRPS operon, and exerts a significant influence on the cytotoxic phenotype of *K. oxytoca*. Transcriptional, in silico, biochemical, and functional analyses collectively support a model in which SdiA enhances *npsA* expression in response to AHL signals. Nevertheless, these findings require cautious interpretation. As direct quantification of TM and TV by LC-MS, HPLC, or related analytical methods was not conducted, the data do not provide definitive evidence that SdiA directly or quantitatively regulates TM/TV biosynthesis. Instead, the results indicate that SdiA modulates the cytotoxicity under the specific experimental conditions tested in *K. oxytoca*.

Transcriptional analyses demonstrated that deletion of *sdiA* significantly reduced *npsA* expression, indicating that SdiA is a positive regulator of *npsA*, the first gene of the NRPS operon. Supplementation with AHL increased *npsA* transcription in the WT strain but failed to affect the ∆*sdiA* mutant, demonstrating that SdiA mediates the transcriptional response to quorum-sensing signals. Importantly, AHL supplementation did not reduce *npsA* expression in the ∆*sdiA* mutant relative to its corresponding untreated condition. Rather, the apparent increase in the expression difference between WT and ∆*sdiA* strains under AHL-supplemented conditions resulted from the selective induction of *npsA* expression in the WT strain.

This behavior is consistent with the established role of LuxR-type regulators, which detect exogenous AHL molecules and modulate gene expression in response to population density and microbial community composition [42,43]. Notably, *K. oxytoca* lacks a LuxI homolog, suggesting that SdiA enables the organism to detect AHL signals produced by neighboring bacterial species and integrate interspecies communication into its regulatory network.

A 10 µM AHL concentration was chosen based on studies showing SdiA-dependent responses in *S. enterica* [20]. The observed changes in *npsA* expression and cytotoxicity indicate activation of the SdiA pathway in *K. oxytoca*. However, this concentration serves only as an in vitro parameter and does not reflect physiological intestinal AHL levels. Micromolar AHL concentrations are commonly used in vitro in quorum-sensing studies with LuxR-type regulators, including SdiA systems, to ensure receptor activation and reproducible transcriptional responses [21,22,23,24,25].

The gastrointestinal tract is a complex and heterogeneous environment in which AHL availability may vary substantially due to microbial composition, spatial organization, diffusion dynamics, oxygen levels, and host-derived factors [11,12,13,14]. Therefore, future dose–response studies using lower AHL concentrations and more physiologically relevant models are required to better define the biological relevance and sensitivity of SdiA-mediated signaling in *K. oxytoca*.

In silico analysis identified a putative promoter upstream of *npsA* and a predicted SdiA-binding site located 71 bp upstream of the putative transcription start site. This regulatory arrangement suggests a role for SdiA in the positive regulation of *npsA* expression. EMSA experiments further supported a direct interaction between purified His_6_-SdiA and *the npsA* upstream regulatory region. Increasing concentrations of purified His_6_-SdiA generated concentration-dependent retardation of the DNA fragment, indicating formation of stable nucleoprotein complexes. The specificity of this interaction was supported by the absence of binding to the negative-control fragment and by strong binding to the *ftsQ* promoter, a previously characterized SdiA target [33].

Collectively, these findings support a direct interaction between SdiA and the *npsA* regulatory region. However, the specific nucleotides required for SdiA-mediated promoter regulation remain unidentified. The lack of mutational validation of the predicted SdiA-binding motif is a significant limitation of this study. Future research should employ site-directed mutagenesis, footprinting analyses, quantitative binding assays, and promoter-reporter systems to confirm the functional binding motif and elucidate how SdiA binding influences *npsA* promoter activity.

EMSA results showed that SdiA binds the *npsA* regulatory region in vitro, both in the presence and absence of exogenous AHL. Comparable results have been reported for other SdiA-regulated promoters [24,33,35,44,45], indicating that exogenous AHL is not strictly required for DNA recognition under in vitro conditions. Although similar DNA retardation patterns were observed in EMSAs, this technique does not provide the quantitative resolution needed to detect subtle differences in binding affinity, protein conformation, or complex stability.

In contrast, RT-qPCR analyses demonstrated increased *npsA* expression in the presence of AHL during bacterial growth, suggesting that AHL may influence SdiA regulatory activity through mechanisms not fully captured by standard EMSA analysis. Further quantitative binding studies are necessary to determine whether AHL affects the affinity, stability, or regulatory properties of the SdiA–DNA interaction.

Functionally, SdiA-mediated regulation significantly influenced the cytotoxic phenotype observed under the tested experimental conditions. Supernatants from the WT strain induced substantial epithelial cell damage, and this effect increased when bacteria were cultured in the presence of AHL, indicating that quorum-sensing signals modulate cytotoxicity-associated activity. In contrast, the ∆*sdiA* mutant exhibited markedly reduced cytotoxicity under both experimental conditions, whereas complementation restored the phenotype in the ∆*sdiA* pT3-SdiA strain.

Importantly, previous studies have demonstrated that *K. oxytoca* strains lacking *npsA* fail to produce TM and TV and concomitantly lose cytotoxic activity toward epithelial cells [6,7,38,46]. For this reason, ∆*npsA* strains are commonly used as controls in studies evaluating the cytotoxicity linked to this genomic region. Consistent with these previous observations, the ∆*npsA* mutant analyzed in the present study exhibited markedly reduced cytotoxicity under the experimental conditions tested.

The cytotoxic phenotype observed in this study is biologically associated with the *npsA*-linked NRPS locus. However, despite this established relationship, definitive conclusions regarding the exclusive or quantitative dependence of cytotoxic effects on TM and TV production cannot be made, as direct toxin quantification was not performed. Consequently, these findings should be interpreted as evidence that SdiA contributes to a cytotoxic phenotype, rather than as definitive proof of direct quantitative regulation of TM/TV biosynthesis.

The potential in vivo relevance of SdiA-dependent regulation should be interpreted with caution. While antibiotic-associated hemorrhagic colitis is an intestinal disease, this study utilized in vitro bacterial cultures and HeLa cell cytotoxicity assays. The gastrointestinal tract is a dynamic and heterogeneous ecosystem in which numerous environmental and microbial factors can influence quorum-sensing responses and *npsA* gene expression. Microbiota-derived AHL molecules can vary greatly in concentration, chemical structure, spatial distribution, and diffusion properties, potentially leading to variable SdiA activation during intestinal colonization [11,12,13,14].

Furthermore, oxygen gradients, nutrient availability, microbial competition, host-derived factors, and spatial heterogeneity within the intestinal niche may also affect *npsA* expression and related cytotoxic phenotypes [47,48]. Thus, the regulatory model proposed here should be viewed as an experimentally supported in vitro framework that requires validation in physiologically relevant systems, such as intestinal colonization and in vivo expression models. Regarding transcriptional regulation, SdiA appears to integrate environmental quorum-sensing signals with the expression of virulence-associated traits in *K. oxytoca*. The observed increases in cytotoxicity and *npsA* expression in the presence of AHL suggest that quorum-sensing molecules modulate the regulatory output of the SdiA signaling pathway during bacterial growth. Similar regulatory patterns have been reported for other LuxR-family proteins, which respond to environmental AHLs and regulate genes involved in host interaction and bacterial adaptation [49,50].

Regulation of the NRPS locus is likely multifactorial and may involve the coordinated action of several global regulatory systems. Previous studies have implicated regulators such as CRP and Lrp in controlling this genomic region [16,40]. Within this broader regulatory network, CRP may link carbon metabolism and cellular energetic status to *npsA* expression, while Lrp may contribute to nutrient-responsive regulation associated with amino acid availability and bacterial physiological adaptation [16,40]. In this context, SdiA may function as an additional quorum-sensing regulatory layer that integrates interspecies signaling information derived from microbiota-associated AHL molecules during intestinal colonization. Rather than acting as an isolated regulator, SdiA likely participates in a broader regulatory framework in which metabolic, environmental, and interspecies signaling pathways converge to modulate *npsA* expression and associated cytotoxic phenotypes.

Comparative analyses across enteric bacteria reveal both conserved and context-dependent aspects of SdiA-mediated regulation. In *S. enterica*, the SdiA regulon is relatively limited and includes genes such as the *pefI-srgC* operon, *srgE*, and the *menFDHBCE* operon [51]. Among these, only *pefI-srgC* has been directly demonstrated to be regulated by SdiA, whereas the remaining loci may be indirectly controlled. These genes are mainly associated with virulence-related functions, including fimbrial regulation and host interaction [42].

In both non-pathogenic and pathogenic *E. coli*, SdiA regulates functionally analogous systems, including the glutamate-dependent acid resistance island (*gad*) and, in enterohemorrhagic *E. coli*, the locus of enterocyte effacement (LEE), the latter through direct regulation of the *ler* promoter [23,45,52]. Additionally, SdiA represses flagellar gene expression, further supporting its role as a regulator that integrates environmental signals with bacterial adaptation [53,54].

In other *Enterobacteriaceae*, SdiA-mediated regulation appears more variable and less well defined. In *Enterobacter cloacae*, the regulon includes genes associated with membrane composition, signal transduction, and metabolism, involving both AHL-dependent and AHL-independent mechanisms [42,55]. Similarly, in *K. pneumoniae*, SdiA has been implicated in regulating *ftsQ*, *rpoS*, and *fimA*, although the extent of AHL dependence remains unclear [44]. Across these genera, SdiA consistently functions as an interspecies signal sensor that modulates genes associated with host interaction, stress adaptation, and virulence-associated traits.

The regulatory architecture described in this study is consistent with a conserved LuxR-type signaling paradigm. Identification of *npsA* as an SdiA-responsive gene in *K. oxytoca* expands the known functional scope of SdiA-mediated regulation while remaining consistent with its broader role as a sensor of interspecies signals and an agent of environmental adaptation.

Although additional regulatory proteins were not directly evaluated, they may cooperate with SdiA to further modulate *npsA* gene expression in response to environmental factors such as oxygen availability, nutrient status, or host-derived signals. Future studies should identify these potential co-regulators and further characterize the regulatory network governing the *npsA* gene expression.

In summary, these findings support a model in which SdiA directly interacts with the *npsA* regulatory region and positively influences *npsA* expression, thereby contributing to cytotoxicity in *K. oxytoca*. However, this proposed regulatory framework remains preliminary and should not be regarded as definitive proof of direct quantitative regulation of tilimycin or tilivalline biosynthesis. Future studies should incorporate direct quantification of TM/TV, mutational validation of the predicted SdiA-binding motif, promoter-reporter analyses, dose–response experiments, and physiologically relevant colonization models. Furthermore, the interplay between SdiA and other global regulators, such as CRP and Lrp, should be further investigated to clarify how metabolic, environmental, and quorum-sensing signals are integrated during *K. oxytoca* colonization and cytotoxicity-associated responses.

## 5. Conclusions

The findings indicate that SdiA acts as a positive regulator of *npsA* expression by directly binding to its upstream regulatory region. SdiA thus mediates quorum-sensing-responsive *npsA* expression and is associated with modulation of the cytotoxic phenotype of *K. oxytoca* under the tested conditions. Evidence from transcriptional, in silico, biochemical, and functional analyses supports this mechanism. The observed increase in *npsA* expression and epithelial cytotoxicity in the presence of AHL further substantiates the role of interspecies quorum-sensing signals in modulating cytotoxicity in *K. oxytoca*.

## Figures and Tables

**Table 1:** Bacterial strains and plasmids used in this study.

**Strains**	Description *	**References**
*K. oxytoca* MIT 09-7231	Wild-type *Klebsiella oxytoca* strain MIT 09-7231, Amp^R^	[15]
*K. oxytoca* ∆*sdiA*	*K. oxytoca* ∆*sdiA::Kan*, Kan^R^	This study
*K. oxytoca* ∆*sdiA* pT3-SdiA	*K. oxytoca* ∆*sdiA::Kan* pT3-SdiA, Kan^R^, Tet^R^ (*trans*-complemented)	This study
*K. oxytoca* ∆*npsA*	*K. oxytoca* Δ*npsA*::FRT	[16]
*E. coli* MC4100	Cloning strain *F^−^ araD139*∆*(argF-lac) U169 rspL150* *relA1 flbB5301 fruA25 deoC1 ptsF25*	[17]
*E. coli* BL21 (DE3)	F^−^ *omp*T *hsd*S_B_(r_B_^−^, m_B_^−^) *gal dcm* (DE3)	Invitrogen
**Plasmids**	Description *	**References**
pKD119	pINT-ts derivative containing the λ-Red recombinase system under an arabinose-inducible promoter, Tet^R^	[18]
pKD4	pANTsy derivative template plasmid containing the kanamycin cassette for λ-Red recombination, Amp^R^ Plasmid that shows temperature-sensitive	[18]
pMPM-T3	p15A derivative low-copy-number expression vector, *lac* promoter, Tet^R^	[19]
pT3-SdiA	pMPM-T3 derivative expressing *sdiA* from the *lac* promoter, Tet^R^	This study
pMPM-T6	p15A derivative expression vector, pBAD (*ara*) promoter, Tet^R^	[19]
pT6-SdiA	pMPM-T6 derivative expressing N-terminal His_6_-SdiA from the pBAD(*ara*) promoter, Tet^R^	This study

* Amp^R^, ampicillin resistance; Kan^R^, kanamycin resistance; Tet^R^, tetracycline resistance.

## Data Availability

The original contributions presented in the study are included in the article/Supplementary Material, further inquiries can be directed to the corresponding author.

## Supplementary Materials

The following supporting information can be downloaded at https://www.mdpi.com/article/10.3390/microorganisms14051144/s1. Table S1: Primers used in this study.

## Abbreviations

The following abbreviations are used in this manuscript:

AAHC	Antibiotic-associated hemorrhagic colitis
TM	Tilimycin
TV	Tilivalline
NRPS	Non-ribosomal peptide synthetase
QS	Quorum-sensing
AHLs	N-acyl homoserine lactones
WT	Wild type
TSB	Tryptic soy broth
OD	Optical density
AHL	N-(3-oxo-octanoyl)-L-homoserine lactone
AmpR	Ampicillin resistance
KanR	Kanamycin resistance
TetR	Tetracycline resistance
RT-qPCR	Reverse Transcription-quantitative PCR
cDNA	Complementary DNA
qPCR	Quantitative PCR
LB	Lysogeny Broth
PBS	Phosphate-Buffered Saline
SDS-PAGE	Sodium dodecyl sulfate-polyacrylamide gel electrophoresis
BSA	Bovine serum albumin
EMSA	Electrophoretic mobility shift assays
TBE	Tris-borate-EDTA
LDH	Lactate dehydrogenase
gad	Glutamate-dependent acid
LEE	Locus of enterocyte effacement
DMEM	Dulbecco’s Modified Eagle Medium
FBS	Fetal bovine serum
ANOVA	Analysis of variance
