# Supplementary material for: The Quorum-Sensing Regulator SdiA Activates npsA Expression and Modulates Cytotoxicity in Klebsiella oxytoca
Source: Microorganisms. 2026 May 19;14(5):1144. doi: 10.3390/microorganisms14051144 (PMC13209580; doi:10.3390/microorganisms14051144)
Supplement: Supplementary file 1 [file microorganisms-14-01144-s001.zip › Table S1.pdf]

Table S1. Primers used in this study*. Sequences are shown in 5’ to 3’ direction.

For qPCR	Target gene	RE
rrsH-F: CAGGGGTTTGGTCAGACACA	*rrsH*	
rrsH-R: GTTAGCCGGTGCTTCTTCTG		
npsA-F: AAATACGTGGCTTCCGCATC	*npsA*	
npsA-R: TCCTGCGTGACATAACAAGC		
**For mutagenesis**		
H1P1-SiA: TCAGGAGTTATTTGGTCAGTTATGAGGGACAATGATTTTT TCAGCTGTAGGCTGGAGCTGCTTCG	*sdiA*	
H2P2-SdiA: CACGCAGAACAACCTATAAGATCAAATTAATCCCGTC GCTACCGCCATATGAATATCCTCCTTAG		
**For characterization of the mutant**		
Kox-SdiA-F: GCCAGTCGCCATCTAATAAATGC	*sdiA*	
Kox-SdiA-R: GTAATGATTCACCGGCTGAAGG		
**For clone *sdiA* into the vector pMPM-T3**		
sdiA-Kpn-T3-F: GGG***GGTACC***GATTATCACTCAGGAGTTATTTGGTCA	*sdiA*	*Kpn*I
sdiA-Bam-T3-R: GGG***GGATCC***GCCATGGGTCAGGCGGAT		*Bam*HI
**For clone *sdiA* into the vector pMPM-T6**		
sdiA-Nco-His-T6-F: GGG***CCATGGCG***CATCATCATCATCATCATAGGGACAATGATTTTTTCAGCT	*sdiA*	*Nco*I
sdiA-Hind-T6-R: GGG***AAGCTT***TCAAATTAATCCCGTCGCTACC		*Hind*III
**For EMSA test probes**		
FtsQP2P1_EMSA_EC_F: CGCAGAGTTTGAAAAAGGCCT	*ftsQ*	
FtsQP2P1_EMSA_EC_R: GCTGTGGCTGGTCATACCC		
npsA-F: TCTCTCACTCGAAATTTAACAGGT	*npsA*	
npsA-R: TCTCTCCTGGAGAATTAGGAACG		

*Underlined text indicates regions of homology to the pKD4 template. Restriction endonuclease (RE) recognition sites are shown in bold italics.
